# Supplementary material for: Alcohol consumption and the risk of gastric intestinal metaplasia in a U.S. Veterans population
Source: PLoS One. 2021 Nov 15;16(11):e0260019. doi: 10.1371/journal.pone.0260019 (PMC8592489; doi:10.1371/journal.pone.0260019)
Supplement: Attachment — Submitted filename: Response to Reviewers.docx [file pone.0260019.s001.docx]

**July 21, 2021**

**Matteo Rota, Ph.D.**

**Academic Editor**

**PLOS ONE**

Dear **Dr. Rota,**

Thank you for considering our manuscript, and for the helpful comments from the reviewers which accompanied your recent letter. We have taken the opportunity of revising our manuscript in accordance with those comments and herewith submit it to you for further consideration.

**Comments from the Editor**

**Comment #1:**

*Estimates were not adjusted for a proxy of socioeconomic status (SES), that is, the main determinants of gastric cancer. This was an important comment from Reviewer #1 that greatly limits your results. Please address this bias.*

**Reply to Comment #1:**

We thank the Editor and Reviewer for this important comment. We captured educational background in our study survey, which we now use here as a proxy for social status. As expected, we show in Table 1 that highest level of education is strongly associated with case-control status (cases are more likely to have high school or less education). We have therefore added highest level of education as a covariate in our multivariable models. Additionally adjusting for education level did attenuate the associations with alcohol consumption to the null. We believe adding this data to the paper strengthens our methods and helps to strengthen confidence in our findings.

**Comment #2:**

*For what regards the dose-risk relationships, the figure appears to depict a linear trend. In fact, when I go through the manuscript text, I found the author’s sentence “There was no evidence for a nonlinear dose relationship (p=0.46 […]”. Therefore, it does not make sense to report a nonlinear trend when there was no evidence against linearity. Therefore, in figure 1 please report a linear relationship and use a logarithm scale for the y-axis. In fact, in the current figure version, the y axis range is inappropriate and not on a log-scale.*

**Reply to Comment #2:**

A common approach to the analysis of alcohol consumption in cancer (and other disease) studies has been to categorize participants based on broad measures of alcohol intake. Such an approach has the advantage of deriving an easily interpretable measure of relative risk, and makes no assumption about any underlying trends or patterns of risk. However, categorization may obscure potentially important differences in risk within and across categories of alcohol intake, particularly if the cut-off points for categorization span biologically important thresholds. Modeling risk with alcohol intake as a continuous measure by using standard linear regression models avoids the potential loss of information owing to categorization, but it constrains the dose effect to be linear. Again, such constraints may mask important differences in risk associated with different levels of alcohol intake. As has been well described, alcohol consumption has a J-shaped relationship with all-cause mortality in human beings and there is no reason to discount the possibility that similar nonlinear patterns of risk may be observed for stomach cancer and its precursor, gastric intestinal metaplasia. In the current manuscript, we use a suite of approaches to address this issue. In total, our analyses consistently demonstrate no association with alcohol consumption. The splines demonstrate no hidden/missing non-linear relationship. The lack of linear association across categories is accurate, and there is no ‘hidden’ patterns of risk obscured by the categorization or assumption of linearity.

**Comment #3:**

*On page 13, the authors wrote “Among alcohol drinkers, the risk for gastric intestinal metaplasia did not increase linearly with increasing total average consumption (p-trend=0.14)”. There is something unclear as it seems that the trend cannot be considered non-linear being the p-value greater than 0.14. Please clarify.*

**Reply to Comment #3:**

There is actually no association with alcohol consumption. The associations with categories of alcohol consumption demonstrated a lack of linear association (no increase with increasing category of exposure). The splines show that there is also no non-linear association. Together, these analyses address issues of categorization and assumptions of linearity. They strengthen our conclusion that alcohol consumption does not increase a person’s risk for gastric intestinal metaplasia.

**Comment #4:**

*Among the limitations, I would state that controls were not matched to cases, as a “natural” consequence of the cross-sectional design.*

**Reply to Comment #4:**

We added the following to the Discussion (version with tracked changes; page 24, paragraph 1):

*“A natural consequence of the cross-sectional design also meant that we could not match controls with cases.”*

**Reviewer #1**

**Comment #1:**

*The authors stated that “participants who reported ever consuming alcohol but who did not consume alcohol at least monthly for ≥6 months were considered non-drinkers and included in the referent group”. An additional analysis in which these individuals are excluded from the referent group is appropriate. Please, make it available.*

**Reply to Comment #1:**

Unfortunately, the alcohol questions in our study survey were written such that we did not capture any alcohol history among persons who may have ever consumed alcohol but who did not consume alcohol at least monthly for ≥6 months. This was the qualifying question for capturing specific alcohol consumption. We have now noted this as a limitation of our study (page 23, paragraph 2):

*“We included in our referent group a fraction of participants who may have ever consumed alcohol but who did not consume alcohol at least monthly for ≥6 months during their lifetime. As a result, we may have biased our risk estimates towards the null.”*

**Comment #2:**

*The authors stated that “The referent group for all analyses was those who were lifelong non-drinkers”. I suppose that ex-drinkers are not included in the referent group. This is correct, but I suggest to clary state this fact.*

**Reply to Comment #2:**

The Reviewer is correct. Ex-drinkers were not included in the referent group. We revised the Methods (page 10, paragraph 2) as follows:

*“For all analyses, we compared history of alcohol consumption among ever drinkers (current and ex-drinkers) to a referent group of those who were lifelong non-drinkers.”*

**Comment #3:**

*No measures/indexes of social status have been included as potential confounders. Due to the high importance that social status has as potential confounder for the association of alcohol intake with health outcome, authors should make any effort to include it in the multivariable model. On the contrary, the absence of any index of social status in the panel of covariates must be discussed as a strong limitation of the manuscript.*

**Reply to Comment #3:**

We thank the Reviewer for this important comment. We captured educational background in our study survey, which we now use here as a proxy for social status. As expected, we show in Table 1 that highest level of education is strongly associated with case-control status (cases are more likely to have high school or less education). We have therefore added highest level of education as a covariate in our multivariable models. Additionally adjusting for education level did attenuate the risk estimates for associations with alcohol consumption to the null.

**Comment #4:**

*Another potentially important variable the authors should adjusted their models for is BMI, also because, beyond formal statistical significance, BMI shows some degree of association with case/control status (Table 1).*

**Reply to Comment #4:**

We agree with the Reviewer and have now adjusted our models for BMI.

**Comment #5:**

*“Missing category was excluded from statistical tests for differences between controls and cases” (Table 1). However, it is not clear how the authors dealt with missing values in multivariable analyses (adjusted also for smoking and HP infection, that have missings). Please clarify. A case complete approach is not appropriate, and some more robust approach (multiple imputation, for example) for dealing with missing values have to be implemented.*

**Reply to Comment #5:**

We apologize for excluding this information from the Methods. As shown in Table 1, we had missing data on BMI, WHR, *H. pylori* infection, and smoking status among a minority of study participants. We added the following to the Methods (page 10, paragraph 2):

*“We included participants with missing data for covariates (e.g., BMI, WHR, H. pylori infection, smoking status) in the analyses using an additional category for missing values.”*

**Comment #6:**

*The authors repeatedly have used terms as “(statistically) significant” or “non-significant”. It appears that some of their conclusions have been based only on statistically significance. This is not correct. Please, avoid it and base your conclusion more on effect size.*

**Reply to Comment #6:**

We have revised our manuscript throughout to remove emphasis on “statistical significance”.

**Comment #7:**

*Authors should provide complete data for biological interaction of H. pylori infection with alcohol consumption on risk of gastric intestinal metaplasia, in the same way they provided in Table 3 data on the association of combined alcohol and smoking exposure.*

**Reply to Comment #7:**

We have now added this data in Table 4 (page 20).

**Reviewer #2**

Title: I would suggest to avoid stating the conclusion (e.g. no association), but to include the study design and study population in the title.

**Reply:** We have changed our title to “Alcohol consumption and the risk of gastric intestinal metaplasia in a U.S. Veterans population”.

Abstract:

1. It was slightly confusing to read the number of cases and number of controls separately in the first place, and later realise that it was a cross-sectional study but not a case-control study. I would suggest to first report the overall sample size recruited, and then report the number of cases identified.

**Reply:** We revised the Abstract (page 3) as follows:

*“We used data from 2084 participants (including 403 with gastric intestinal metaplasia) recruited between February 2008-August 2013 into a cross-sectional study at the Michael E. DeBakey Veterans Affairs Medical Center in Houston, Texas.”*

2. Please state the full name of Houston VA Medical Center to make it clear the study population was among veterans.

**Reply:** We replaced “Houston VA Medical Center” with “Michael E. DeBakey Veterans Affairs Medical Center in Houston, Texas”.

3. Please state the study period (in Abstract and also in the Methods later).

**Reply:** We added the study period to both the Abstract (page 3) and Methods (page 7).

Methods:

1. There was no mentioning of missing data until missing data was shown in Table 1. Please indicate in the Statistical analysis the number of participants with missing data and how missing data was handled in each of the subsequent analyses.

**Reply:** We apologize for excluding this information from the Methods. As shown in Table 1, we had missing data on BMI, WHR, *H. pylori* infection, and smoking status among a minority of study participants. We added the following to the Methods (page 10, paragraph 2):

*“We included participants with missing data for covariates (e.g., BMI, WHR, H. pylori infection, smoking status) in the analyses using an additional category for missing values.”*

2. Page 7, please move the sentence “Overall, 70% of patients in the elective EGD …” up to the end of the Study population paragraph on page 6.

**Reply:** We moved this sentence as suggested.

3. How many participants were recruited from each of the elective EGD group and of the primary care group, respectively?

**Reply:** We added this information to the Results (page 12, paragraph 1):

*“This study included data from 2084 participants, with 1568 recruited from EGD clinics and 516 from primary care.”*

4. Please specify how much one drink quantifies (e.g., in grams or units).

**Reply:** We now noted in the Methods that one standard drink was 10g of alcohol.

Results:

1. Page 11, please report the overall sample size and provide a summary of descriptive data of the whole study population in the beginning, before moving on to describe cases and controls separately.

**Reply:** We revised the Results (page 12, paragraph 1) as follows:

*“This study included data from 2084 participants, with 1568 recruited from EGD clinics and 516 from primary care. The mean age of participants was 60.2 years (standard deviation, 8.1 years). Ninety-two percent of participants were male, 57.3% White and 31.3% Black. Most participants were overweight or obese (81.4%) and reported a history of smoking (68.5%).”*

2. Table 1 title: The terms case and controls are self-explanatory, so there is no need to repeat “controls without” and “cases with gastric intestinal metaplasia”. I would suggest to change the title along the lines of “Characteristics of cases and controls” or “Characteristics of US Veterans by case status”

**Reply:** We changed the title to “Characteristics of cases and controls.

3. Table 1: Please indicate the method used to derive the p-values.

**Reply:** We added this to the footnote of Table 1.

4. Page 13, please report IQR for median of alcohol intake.

Reply: We revised the results (page 15, paragraph 1) as follows:

*“Among controls who reported being ever drinkers, the median lifetime total alcohol consumption was 13 (interquartile range [IQR], 6-42) and 4 (IQR, 2-10) drinks/week for males and females, respectively. Median lifetime total alcohol consumption was numerically higher among male (16 drinks/week; IQR, 6-42) and female (8 drinks/week; IQR, 2-14) cases with non-cardia gastric intestinal metaplasia than controls.”*

5. Table 2: Please be consistent with terminology e.g. non-drinkers (table and main text) vs. never drinkers (footnote).

**Reply:** We placed “never drinkers” with “non-drinkers” in the footnote (page 17).

6. Figure 1 title: Please remove “compared with controls” from the title.

**Reply:** We removed “compared with controls” from the figure title.

Discussion

1. The authors briefly mentioned generalizability of the study population (predominantly white males) as a limitation. However, other aspect of the study population (Veterans only), as well as the different response rate between the elective EGD group and the primary care group (if the socio-demographics or health seeking behaviours differ between the two groups), may also have implications on the generalizability to the general population and bias. It would be good to see some discussion in the paper on these points.

**Reply:** We added the following to the Discussion (page 23, paragraph 2):

*“The overall response rate among the primary care group was 43% and this may have biased our results. However, participants who consent are generally healthier than the general population and, if one existed, this would strengthen an association.”*

2. Please discuss the potential bias of self-reported alcohol intake in the limitations.

**Reply:** We added the following to the Discussion (page 24, paragraph 1):

*“Additionally, the risk estimates may be subject to reporting bias due to self-report alcohol intake. We attempted to limit this bias by having participants complete the study surveys prior to the study EGD (and knowledge of case-control study), increasing the likelihood that any bias would be non-differential between cases and controls.”*

3. Diet is an important risk factor for stomach cancer but was not measured in the study – this should be discussed in the limitation.

**Reply:** We added the following to the Discussion (page 24, paragraph 1):

“Diet is an important risk factor for gastric cancer; however, we did not include diet in our study, which may have confounded our associations with alcohol consumption.”

4. Page 20, final sentence “alcohol use to any degree alone proved to be an insufficient predisposing factor”. The word “proved” seems a bit too strongly conclusive, especially in the context of cross-sectional study - a more suggestive tone would be more appropriate.

**Reply:** We revised the sentence (page 24, paragraph 2) as follows:

*“While there was some evidence of an additive effect between tobacco smoke and alcohol consumption on non-cardia gastric intestinal metaplasia risk, alcohol use to any degree alone may not confer increased risk for gastric intestinal metaplasia.”*

**Journal Requirements:**

When submitting your revision, we need you to address these additional requirements.

1. Please ensure that your manuscript meets PLOS ONE's style requirements, including those for file naming.

**Reply:** We have edited our files according to the journal templates.

2.  Please include in your Methods section the date ranges over which you recruited participants to this study.

**Reply:** We have included the date range in the Methods (page 7).

3. We note that you have indicated that data from this study are available upon request. PLOS only allows data to be available upon request if there are legal or ethical restrictions on sharing data publicly. For information on unacceptable data access restrictions, please see http://journals.plos.org/plosone/s/data‐availability#loc‐unacceptable‐data‐accessrestrictions.

**Reply:** There are legal restrictions on sharing data publicly. The dataset used for this study contains sensitive patient information that is potentially identifying. The Department of Veterans Affairs (VA) restricts access to such data unless researchers meet specific criteria per VHA Directive 1605.01, Privacy and Release of Information, section 13e which includes a written request for the data, IRB approval with waiver for HIPAA authorization prior to the request for individually identifiable information, and approval by the VA. These national sharing policies and standards also apply to deidentified data. Data requests may be sent to: Center for Innovations In Quality, Effectiveness, and Safety (IQuESt) 2450 Holcombe Blvd Suite 01Y Houston, TX 77021 713-791-1414 Attn: Laura Petersen, MD, MPH MEDVAMC Associate Chief of Staff, Research; Director IQuESt. [Laura.Petersen@VA.gov](mailto:Laura.Petersen@VA.gov)

We look forward to your consideration of our revised manuscript, and trust that these revisions meet with your approval.

Yours sincerely,

Aaron P. Thrift, PhD

*On behalf of all authors*

Associate Professor

Section of Epidemiology and Population Sciences

Department of Medicine

Baylor College of Medicine
