# Supplementary material for: Alcohol consumption and the risk of gastric intestinal metaplasia in a U.S. Veterans population
Source: PLoS One. 2021 Nov 15;16(11):e0260019. doi: 10.1371/journal.pone.0260019 (PMC8592489; doi:10.1371/journal.pone.0260019)
Supplement: Attachment — Submitted filename: Response to Reviewers.docx [file pone.0260019.s002.docx]

**October 20, 2021**

**Matteo Rota, Ph.D.**

**Academic Editor**

**PLOS ONE**

Dear **Dr. Rota,**

Thank you for considering our revised manuscript, and for the additional comments in your recent letter. We have taken the opportunity of revising our manuscript in accordance with those comments and herewith submit it to you for further consideration.

**Comments from the Editor**

**Comment #1:**

*There are still two minor comments that need to be better addressed before taking a positive decision on the manuscript. See below. This is a minor revision. In addition to these reviewer #2 comments, I would ask to the authors to better set the y-axis limits of Figure 1, now ranging from 0.1 to 10. I suggest to set a range from 0.25 to 4, using a logarithmic scale, as common for plots of dose-risk relationships.*

*I also want to point out that my previous comment #2 was partly misunderstood by the authors. The assessment of dose-risk relationship shape trough restricted cubic spline (rcs) showed no evidence of a non-linear association. This is fine, but from a statistical point of view, this means that there is no reason to consider a less parsimonious model (as the rcs is) when no evidence of a better fit emerged as compared to the linear line. However, I still agree with the author choice to show the rcs dose-risk relationship, but I also suggest to report the linear line in a supplementary figure, if not possible within Figure 1.*

**Reply to Comment #1:**

We apologize for our misunderstanding your prior comment. We have revised the Figure to set the y-axis limits as suggested and added a linear line. We hope that this new Figure better reflects the findings from our study.

**Reviewer #2**

*The authors have generally done well in revising the manuscript and responding to the reviewers’ comments. However, I still have a few minor comments:*

**Comment #1:**

*Limitation paragraph, discussion on self-report bias in alcohol consumption: even if the bias in exposure status is non-differential between cases and controls, non-differential misclassification of the exposure can still bias the association toward the null.*

**Reply to Comment #1:**

Thank you for this important comment. We have revised the Discussion (page 22, paragraph 1) as follows:

*“Additionally, the risk estimates may be subject to reporting bias due to self-report alcohol intake. Although we attempted to limit this bias by having participants complete the study surveys prior to the study EGD (and knowledge of case-control study), and the bias in exposure status would likely be non-differential between cases and controls, it may have biased the association toward the null.”*

**Comment #2:**

*Limitation paragraph, discussion on diet: The first half of the sentence “Diet is an important risk factor for gastric cancer; however, we did not include diet in our study…” sounds fine as poor diet/nutrition is associated with heavy drinking, and is a possible confounder in any observed association between alcohol consumption and gastric cancer-related outcome. However, the second part of the sentence “… which may have confounded our associations with alcohol consumption” is a bit be confusing in this context since there is no observed association between alcohol consumption and risk of gastric intestinal metaplasia in this study. I would suggest the authors to revise the second part of sentence to discuss this issue more specifically in the context of this study. For example, the lack of diet info in this study is unlikely to have major impact on your conclusions (since* *as a confounder diet would have exaggerated the observed associations, instead of biasing it toward the null).*

**Reply to Comment #2:**

We have revised the sentence (page 22, paragraph 1) as follows:

*“Diet is an important risk factor for gastric cancer; however, we did not include diet in our study. Nonetheless, lack of adjustment for diet would not explain the null association because, as a confounder, diet would instead have exaggerated an observed association, instead of biasing it toward the null.”*

We look forward to your consideration of our revised manuscript, and trust that these revisions meet with your approval.

Yours sincerely,

Aaron P. Thrift, PhD

*On behalf of all authors*

Associate Professor

Section of Epidemiology and Population Sciences

Department of Medicine

Baylor College of Medicine
